# Applying mass spectrometry based proteomic technology to advance the understanding of multiple myeloma

**DOI:** 10.1186/1756-8722-3-13

**Published:** 2010-04-07

**Authors:** Johann Micallef, Moyez Dharsee, Jian Chen, Suzanne Ackloo, Ken Evans, Luqui Qiu, Hong Chang

**Affiliations:** 1 Department of Laboratory Hematology, University Health Network, 200 Elizabeth Street, Toronto, M5G-2C4, Canada; 2 Department of Laboratory Medicine and Pathobiology, University of Toronto, 1 King's College Circle, Toronto, M5S-1A8, Canada; 3 Ontario Cancer Biomarker Network, MaRS Centre, South Tower, Suite 200, 101 College Street, Toronto, M5G-1L7, Canada; 4 Department of Hematology and Oncology, Institute of Hematology & Blood Diseases Hospital 288 Nanjing Road, Tianjin 300020, China

## Abstract

Multiple myeloma (MM) is the second most common hematological malignancy in adults. It is characterized by clonal proliferation of terminally differentiated B lymphocytes and over-production of monoclonal immunoglobulins. Recurrent genomic aberrations have been identified to contribute to the aggressiveness of this cancer. Despite a wealth of knowledge describing the molecular biology of MM as well as significant advances in therapeutics, this disease remains fatal. The identification of biomarkers, especially through the use of mass spectrometry, however, holds great promise to increasing our understanding of this disease. In particular, novel biomarkers will help in the diagnosis, prognosis and therapeutic stratification of MM. To date, results from mass spectrometry studies of MM have provided valuable information with regards to MM diagnosis and response to therapy. In addition, mass spectrometry was employed to study relevant signaling pathways activated in MM. This review will focus on how mass spectrometry has been applied to increase our understanding of MM.

## Multiple Myeloma

Multiple myeloma (MM), the second most common blood cancer in adults, is a neoplasm of terminally differentiated B-cells characterized by clonal expansion of malignant plasma cells in the bone marrow. The most common symptoms associated with MM include lytic bone lesions, renal failure, calcium dysregulation, anemia and susceptibility to infections. The median age at diagnosis of MM is 62 years for men and 61 years for women, with less than 2% of those diagnosed at an age less than 40 years. The incidence of MM in the USA is more common among men (7.1 per 100,000) than women (4.6 per 100,000). In addition, MM is two times more frequent in the black population than in the white population [1]. Despite advances in clinical care, MM remains an almost universally fatal disease with a median survival of 3-4 years following conventional treatment and 5-7 years with high dose therapy followed by autologous stem cell transplantation [1].

The development of MM constitutes a series of progressive genetic events. A seminal event is the inappropriate translocation of oncogenes from partner chromosomes into the immunoglobulin heavy chain switch region (IgH) locus on chromosome 14q32. In the past several years, five recurring translocation partners have been defined and mapped to the earliest stages of the developing MM clone [2-4]. The translocations involve partner oncogenes cyclin D1 (11q13), cyclin D3 (6p21), fibroblast growth factor receptor 3 (FGFR3, 4p16), c-maf (6q23) and mafB (20q11). These recurrent translocations are identified with high frequency in primary patient samples and, between them, are found in approximately 50% of MM [5,6]. The remaining 50% of MM lack translocations and are characterized by chromosomal duplication (hyperdiploidy) with associated up-regulation of cyclins D1, D2 and D3 although the molecular pathogenesis is unclear [5]. An equally early event in the genesis of MM appears to be loss of part of chromosome 13 at 13q14.3, although the specific tumor suppressor gene(s) in this region have yet to be identified [7,8]. These events all occur early in disease onset and are often present during an asymptomatic and stable form of the disease called monoclonal gammopathy of unknown significance or MGUS. Active disease must therefore require subsequent genetic events such as mutation/deletion of *p53 *or *Ras *mutations [9].

We have evaluated the prognostic significance of recurrent genomic aberrations including del(13q), t(11:14)/CyclinD1, t(4;14)/FGFR3, t(14;16)/c-Maf, del(17p)(p53), 1q21(CKS1B) amplification, 1p21/CDC14a deletion, and PTEN deletions, as well as CD56 expression in large cohorts of MM patients uniformly treated at our center [10-26]. In addition we have evaluated the impact of chromosomal aberrations on MM patients receiving novel therapies such as the proteasome inhibitor, bortezomib or the immunomodulatory drug lenalidomide. While high-risk genetic factors (t(4;14, del(17p)(p53) deletion, or 13q deletion) did not affect the response or survival of refractory/relapsed MM patients treated with bortezomib [27], del(17p)(p53) deletion had a negative influence on progression free and overall survival of MM patients receiving lenalidomide and dexamethasone [28].

In addition to the cytogenetic studies which have given us significant insight into MM diagnosis and prognosis, gene-expression profiling of MM has also significantly contributed to our understanding of this disease. Due to the highly heterogeneous nature and complexity of MM, gene expression profiling is well suited to study this cancer as it allows for the identification and differentiation of hundreds of genes between various disease states. Several groups have used gene expression arrays, for example, to evaluate drug response in MM patients. Mulligan *et al*. identified a pretreatment expression pattern and predictive markers that could differentiate between bortezomib and dexamethasone response [29]. Other groups have used expression arrays to identify genes involved in doxorubicin and dexamethasone resistance in MM [30,31]. Gene expression arrays have also been used to determine the genetic differences between plasma cells and MGUS and MM cells [32]. These studies have made significant contributions to our understanding of the molecular development as well as mechanisms of drug resistance of MM.

A complementary approach to the study of gene expression profiling is proteomic profiling. The advantages of this approach, which has been increasing in popularity over the past several years, is the ability to determine protein expression levels, post-translational modifications and protein-protein interactions, all of which may have a direct consequence to cell function; such information cannot be obtained through gene expression profiling. Furthermore, several studies found that there is not a significant overlap between gene and protein expression profiles [33-35]. Therefore direct approaches to studying the protein profile of MM are necessary.

Traditional methods to study proteins, such as western blot analysis or immunohistochemistry, have their shortcomings as high throughput solutions for protein profiling including the need for large amounts of tissue as well as the availability of well-characterized antibodies. Mass spectrometry techniques, on the other hand, offer a robust, high throughput method that overcomes many of these limitations [36]. Most important to cancer research, mass spectrometry can be employed to identify known and novel differentially expressed proteins between different tumor samples. This would allow for the identification of biomarkers that can be used in diagnosis, prognosis, and treatment assessment.

## Mass Spectrometry

**Figure 1:**
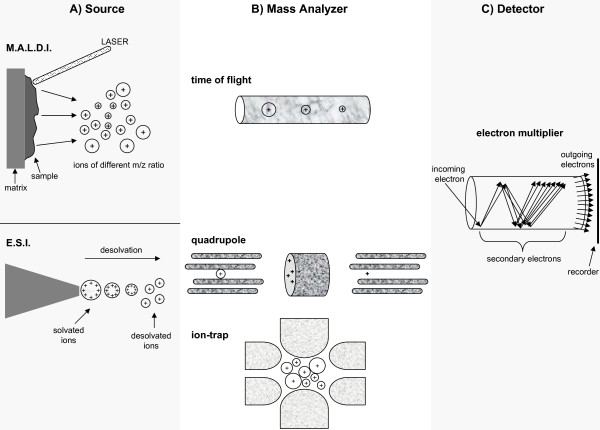
**The mass spectrometer**. (A) Source. In ESI a liquid containing a protein/peptide mixture is passed through a high-voltage capillary tube resulting in charged peptides. In MALDI, a laser is used to excite a chemical matrix containing peptides leading to ejection of charged peptides into the gas phase. (B) Mass analyzers. The quadrupole uses both AC and DC current to affect the trajectory of incoming charged particles. The first quadrupole acts as a mass filter allowing only certain ions to pass into the second quadrupole, the collision cell, where they collide with a neutral gas, undergo fragmentation and enter into the third quadrupole that also acts as a mass filter. The ion-trap mass analyzer uses an AC voltage to "trap" ions. By increasing the AC amplitude, ions of increasing m/z ratio are ejected and measured by the detector. In Time of Flight (TOF), ions of different m/z values are injected into one end of the tube so that they each have approximately identical kinetic energy as they accelerate through the tube. Ions of less mass will reach the detector faster than those that are heavier. (C) Detector. As an ion strikes the surface of the electron multiplier detector, it causes the emission of electrons, which in turn results in the release of secondary electrons. This multiplication process results in the generation of 100 million electrons per incident ion. The arrival of the electron pulse registers as a single ion count.

A mass spectrometer determines the mass of a molecule by measuring its mass-to-charge ratio (m/z). Each mass spectrometer consist of three components; i) the source, which generates ions from a sample either by matrix-assisted laser desorption ionization (MALDI) or electrospray ionization (ESI), ii) a mass analyzer, which resolves peptide ions according to their m/z ratio, and iii) a detector which determines ion abundance for each corresponding ion resolved by the mass analyzer according to their m/z value and generates a mass spectrum (Figure 1). Depending on the type of mass spectrometer used, peptide mass (MS) and/or peptide sequence (MS/MS) data can be obtained from the mass spectrum. This information is then used to search public databases for protein identification such as those maintained by the National Center for Biotechnology Information (NCBI) and the European Bioinformatics Institute (EBI).

## Mass Spectral Analysis

Biological samples including cell lines maintained in culture, biopsy specimens, and serum are very complex in nature as they contain not only an abundance of proteins, but also a large amount of lipids and nucleic acids [36]. Although the mass spectrometer is capable of resolving complex mixtures, protein identification can be greatly simplified if this complexity is reduced. Biological samples are typically lysed with detergents that solubilize proteins, separating them from lipids and nucleic acids. Subsequent procedures can then be employed to further simplify the protein mixture. One dimensional gel electrophoresis can be used to separate proteins according to their molecular weight. Alternatively, two-dimensional gel electrophoresis can be used to achieve greater protein separation by resolving proteins according to their isoelectric value (pI) and molecular weight. Following electrophoresis proteins are stained with dyes such as Coomassie blue, excised, digested "in-gel" into peptides and then analyzed by the mass spectrometer. Although gel electrophoresis is capable of reducing the complexity of a mixture it has its limitations [37]. Most notably, gel electrophoresis has a limited dynamic range of detection as protein bands are excised only if they can be visualized following staining. The level of detection by MS, however, is below the level of detection of staining and hence many relevant proteins may be missed. A further disadvantage of 2D separation is that it is often difficult to reproduce and some proteins cannot be resolved according to their pI value [37].

An alternative method to reduce the complexity of a protein mixture is the use of liquid chromatography (LC) [36]. Typically, proteins are first digested into peptides and then resolved by LC. The separation of peptides is usually achieved according to charge and molecular weight. Often, the peptides that are resolved by LC are directly analyzed by the mass spectrometer. The main advantage of LC is that this method avoids the low dynamic range limitation encountered by gel staining.

### Quantitative Proteomics

In order to improve the diagnosis, prognosis and treatment stratification of those afflicted by cancer the identification of biomarkers indicative of these parameters are necessary. Quantitative protein analysis by mass spectrometry in which a tumor cell may be compared to a normal cell or a drug resistant tumor cell is compared to a drug sensitive tumor cell, provides an effective way to discover these biomarkers. There are two main methodologies to quantify proteins within a sample, stable isotope labeling and label free methods. Both these techniques have been widely used for biomarker discovery [38-45].

One of the most commonly used stable isotopes is the isobaric tags for relative and absolute quantification (iTRAQ). The iTRAQ method allows the simultaneous comparison of up to 8 different samples. The iTRAQ reagent labels the N-terminus of tryptic peptides as well as the amino group side chain of lysine residues [36]. Proteins from different samples are first digested to yield peptides. Each peptide sample is then labeled with one of the iTRAQ reagents. Each reagent consists of i) a reporter group with a molecular weight of 113, 114, 115, 116, 117, 118, 119, or 121 Da; ii) a linker group that also varies in molecular weight to 'balance' the difference of the reporter group; and iii) a peptide reactive group that reacts with the N-terminus of peptides and lysine side chains. Labeled samples are then mixed together and analyzed by the mass spectrometer. Collision induced dissociation of iTRAQ-labeled peptides generates sequence information as well as relative quantification data between the samples.

Recent trends in discovery proteomics are now inclined towards using label-free relative quantification based on the linear relationship between sampling statistics observed using LC-MS/MS and relative protein abundance [46]. Sampling statistics evaluated as potential measures of relative protein abundance include 1) the mean peak area intensity of all peptides identified for an individual protein in a complex sample [47]; 2) the peptide count, or total number of peptides identified from a given protein in a LC-MS/MS experiment [46,48]; and 3) spectral counts, or the total number of tandem mass spectra generated on a given peptide in an LC-MS/MS experiment [47,49-52].

The use of label free techniques has several advantages [53]. First it is more cost effective and less time consuming compared to labeling methods since the labeling reagents do not have to be purchased and experiments to incorporate the stable isotopes into samples are bypassed. Label free methods therefore require less sample modification and avoid increasing sample complexity associated with mixtures of tagged peptides. A second advantage of label free methods is that theoretically there is no limit to the number of samples that can be compared whereas with isotope labeling such as iTRAQ a maximum of 8 samples can be compared at a time. Another advantage of the label free method is that it may provide a higher dynamic range in terms of quantification, although this comes at the expense of unclear linearity and relatively low accuracy [53]. Although there are several advantages to label free methods, it is essential that these methods are robust and reliable in order to control for any experimental variables and that sample processing does affect the outcome of analyses [54].

## Mass Spectrometry to study Multiple Myeloma

### Serum markers for MM diagnosis and prognosis

One of the greatest challenges we face in the clinical setting is the development of tests that would allow for the early detection of cancer. It is well accepted that the earlier tumor cells are detected, the better the prognosis. Certain cancers, including breast, colon and prostate can be detected at an early stage through routine physical exams. For example, screening for prostate specific antigen (PSA) may be useful for the early detection of prostate cancer. Unfortunately, there are no reliable biomarkers that can be used for the early detection of MM and patients are often diagnosed after presenting with clinical manifestations.

A recent study has found that virtually all cases of MM arise from MGUS [55]. On the other hand, the majority of MGUS cases will not develop into MM. Although the status of M-protein may offer insight into the development of MM, it is not absolute, and thus there is a need to identify biomarkers that can predict progression to MM in patients diagnosed with MGUS.

Several groups have been using mass spectrometry based techniques in order to identify potential biomarkers that are early predictors of cancer development [56-61]. Elucidation of these early biomarkers for various cancers, including MM, would be most easily identified from plasma or serum. The advantage of screening blood is that it is easily obtained and contains a large amount of proteins which increase the likelihood of biomarker discovery [62]. One strategy for the early detection of cancer relies on the immune response, which is believed to make auto-antibodies against cancer cells and because the immune response involves an amplification process, these antibodies may be present in sufficient quantities for detection [63,64]. Regardless of the type of biomarker, it will be essential that they are both tumor specific and tissue specific so that the identification and location of the tumor can be determined. Because an overlap most likely exists in biomarker expression between different tumor types, a panel of biomarkers would have to be identified rather than relying on a single protein.

In addition to the identification of early biomarkers that can predict MM, it is also clinically relevant to identify markers that are used for the diagnosis and prognosis of MM. Currently these include calcium, creatinine, hemoglobin, albumin, beta2-microglobulin and monoclonal antibodies. In addition, disease relapse can be monitored by assessing the levels of monoclonal antibodies including heavy chains as well as κ and λ light chains. Koomen's group is currently developing mass spectral techniques that will allow for the quantitative detection of immunoglobulin associated peptides [65]. As MS analysis within the clinical setting becomes more accepted and affordable, successful development of these tests could offer advantages over current clinical tests that are more qualitative in nature, slower, and of lower throughput [65].

Several groups are using mass spectrometry to identify additional biomarkers that may allow for a more specific and sensitive method to diagnose MM. Wang *et al*. employed MALDI-TOF-MS and identified a panel of three biomarkers that correctly identified 26 out of 30 (87%) MM patients and 34 out of 34 (100%) of all normal donors [66]. However, these markers were unable to differentiate between MM and other plasma cell dyscrasias including MGUS, Waldenstrom's macroglobulinemia, solitary plasmacytoma, as well as other tumors with osseus metastasis. Therefore, as the authors mention, it will be necessary to increase their samples size in order to identify additional markers that may unequivocally identify MM patients. Nevertheless this work demonstrates the usefulness of MALDI-TOF MS for the identification of novel biomarkers.

Another group also used mass spectrometry to identify serum biomarkers that might discriminate between patients with skeletal involvement [67]. This group screened serum samples from 48 patients either with evidence of skeletal involvement (24 patients) or without evidence of skeletal involvement (24 patients). Using a partial least squares discriminant analysis (PLS-DA), and a non-linear, random forest (RF) classification algorithm, they were able to predict skeletal involvement with an accuracy between 96-100% using the PLS-DA model and obtained a specificity and sensitivity of 87.5% each with the RF model based on four peaks. Although this study demonstrates the usefulness of proteomic profiling in the diagnosis and treatment of MM progression, further validation studies in additional patient samples are needed.

### Proteins that confer drug resistance in Multiple Myeloma

As mentioned earlier, MM remains a largely incurable disease despite a plethora of chemotherapeutic drugs. This is mainly due to the acquisition of drug resistance by tumor cells. The molecular mechanisms responsible for drug resistance are not well understood. Moreover, it is likely these resistance pathways are unique for each drug. Two scenarios can be envisioned in the acquisition of drug resistance. First, tumor cells may express proteins prior to drug treatment that will render them resistant, and second, tumor cells may acquire resistance following drug administration. An understanding of the molecular signatures that confer drug resistance will be of significant benefit in treatment stratification and will enable the design of novel therapeutic strategies.

#### Bortezomib

Bortezomib, a proteasome inhibitor, has been approved for the treatment of MM patients who have received at least two prior therapies and progressed during the last treatment [68-70]. This drug has been shown to induce apoptosis in various cancer cells, including MM and other lymphomas. It also affects nuclear factor-kB (NF-kB), the bone marrow microenvironment and various cytokine interactions, including, IL-6 [68-70]. Despite significant benefits with regards to time to progression, overall survival and a trend to a lower incidence of infections >grade 3, bortezomib induced an overall response rate of only 35% in refractory and relapsed MM patients (pivotal phase-II (SUMMIT) trial) [68]. In order to determine if recurrent molecular cytogenetic changes identified in MM contribute to the response of bortezomib therapy, we used fluorescence *in situ *hybridization combined with cytoplasmic immunoglobulin light chain stainings (cIg-FISH) and found that the response to bortezomib was independent of recurrent genomic aberrations in MM patients [27]. These observations were confirmed by two independent research groups [71,72].

**Figure 2:**
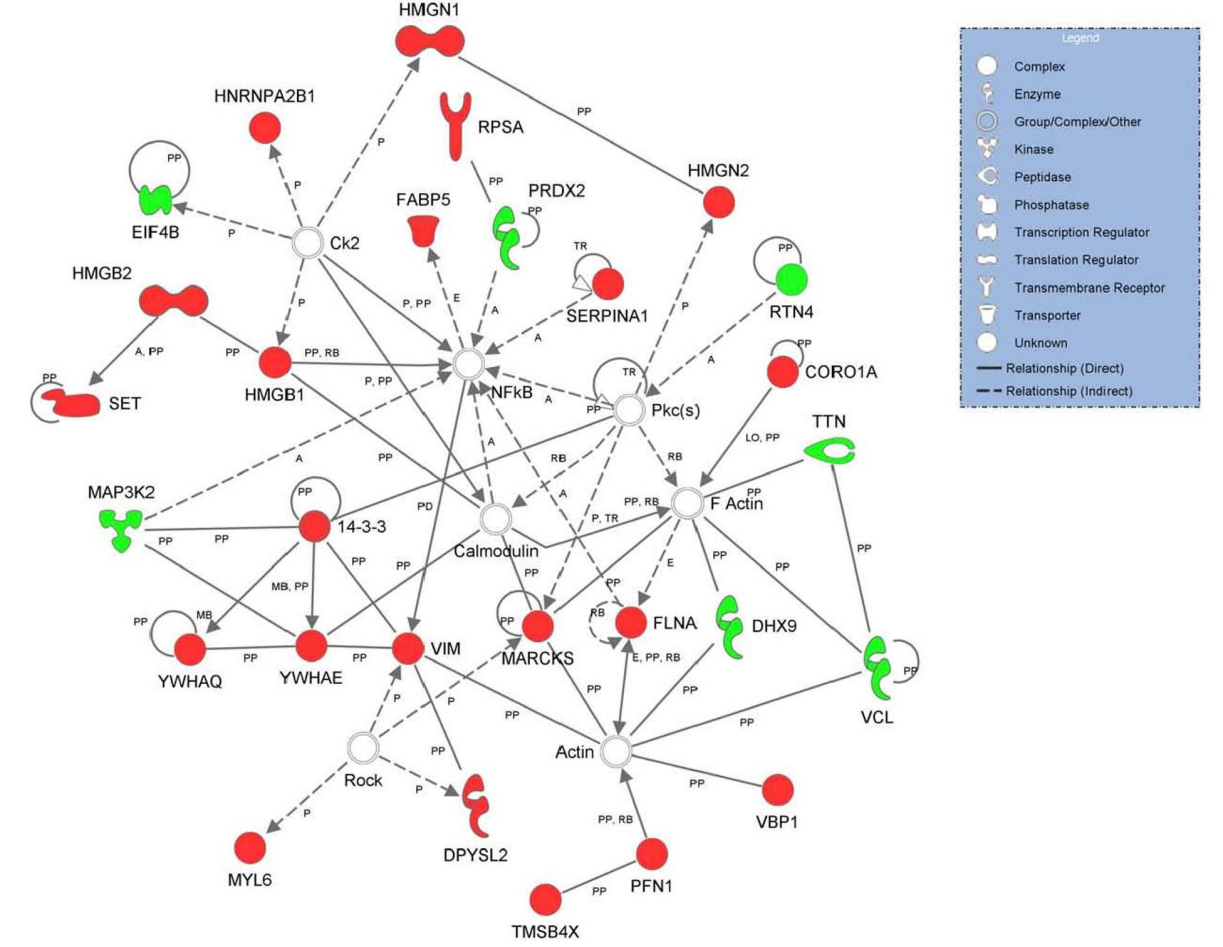
**Highest scoring molecular interaction network generated from Ingenuity Pathways Analysis (IPA) software**. Top functional annotations associated with this network were "Cancer", "Cellular Assembly and Organization", and "Cellular Function and Maintenance". Up-regulated (red) and down-regulated (green) proteins in the 8226/R5 cell line detected in the iTRAQ-MS study are connected by additional protein interactors (white). Both direct and indirect interactions are shown (solid and dashed lines, respectively) with arrows indicating the direction of the underlying relationship, where applicable; types of interactions include activation (A), expression (E), localization (L), membership (MB), phosphorylation (P), protein-DNA (PD), protein-protein (PP), regulation of binding (RB), and translocation (TR). Network analysis was performed on differentially expressed proteins using the Core Analysis feature in IPA version 6.2, and the following analysis settings: data source: Ingenuity Expert Findings; species: human, mouse, rat, uncategorized; tissues and cell lines: all selected.

In light of the above observations, our group is taking a proteomic based approach in order to identify biomarkers that may predict and contribute to bortezomib resistance. To undertake this study we have used iTRAQ analysis to identify differentially expressed proteins between the 8226/R5 bortezomib resistant multiple myeloma cell line and the 8226/S bortezomib sensitive multiple myeloma cell line. Using this approach we identified 30 proteins that were either significantly up or down regulated in the 8226/R5 cell line compared to the 8226/S cell line [73]. Biological systems analysis of these putative markers using Ingenuity Pathway Analysis software revealed that they were associated with cancer-relevant networks (Figure 2). Of particular interest is the MARCKS protein which we found to be over-expressed in the 8226/R5 cell line. MARCKS is a PKC substrate protein that has been found to be over-expressed in several cancers, including glioblastoma multiforme where it was shown to play a role in glioma cell invasion [74]. We have shown that MARCKS is over-expressed in 9 (50%) of 18 multiple myeloma cell lines. In addition, a preliminary screen of pre-bortezomib treatment MM patient samples by immunohistochemistry showed over-expression of MARCKS is associated with bortezomib resistance. We are currently evaluating whether MARCKS plays a role in drug resistance and/or contributes to other tumorgenic properties of MM.

**Figure 3:**
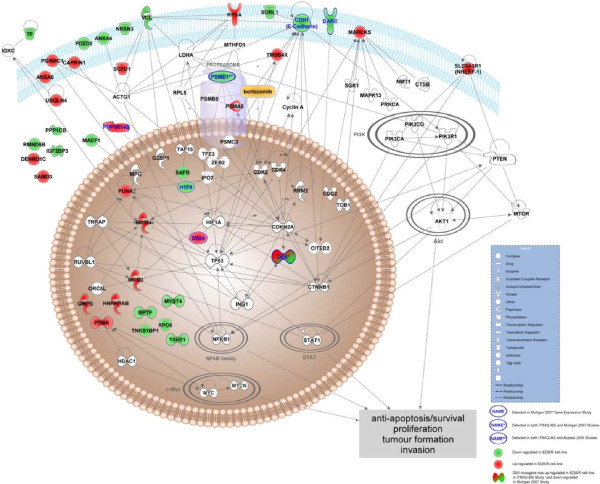
**Comparison of protein and gene expression studies**. Interaction network diagram combining a subset of differentially expressed proteins detected in iTRAQ-MS pilot study, and gene products from two microarray-based gene expression studies investigating bortezomib resistance [29,89]. Up-regulated (red) and down-regulated (green) proteins in the 8226/R5 cell line from each of the three studies are connected by intermediate interactors (white). Expression of DEK oncogene was observed in both iTRAQ-MS and Mulligan [29] studies; proteasome (prosome, macropain) 26S subunit non-ATPase 1 (PSMD1) was expressed in both iTRAQ-MS and Buzzeo [89] studies. Integration of the pilot proteomics data with gene expression datasets indicates complimentarity at the protein interaction and pathway level. Differentially expressed proteins (fold-change = 1.5) measured by iTRAQ-MS are shown to interact directly with a number of oncogenic signaling molecules including TP53, c-Myc, NF-kB, STAT, and PI3K, suggesting possible roles as upstream effectors or indicators of anti-apoptotic and/or tumorgenic processes. Other direct interactors of measured proteins include therapeutic targets in multiple myeloma, including PSMB5 (bortezomib), CDK2 (flavopiridol), and RRM2 (fludarabine phosphate). Protein interactions and illustration were generated with Ingenuity Pathways Analysis version 8.0-2602. Protein interactions were restricted to direct types (default selections) with the term "cancer" as a disease annotation in human/mouse/rat and in uncategorized species.

Protein expression data obtained from our iTRAQ analysis comparing 8226/R5 versus 8226/S cell lines was also compared with gene expression array data from the literature that contrasted MM bortezomib resistant to bortezomib sensitive cells. As expected, there was minimal overlap between these datasets. However these lists of genes and proteins showed strong complementarity in terms of the functional and biological systems with which they are associated, suggesting the systems affected by them or those which they affect may be closely inter-related **(**as illustrated by the network diagram in Figure 3). These data demonstrate the usefulness of proteomic profiling over conventional gene array approaches.

Recently, Hsieh *et al*. used mass spectrometry to identify early biomarkers of bortezomib resistance from the serum of MM patients [75]. They found both apolipoprotein C-I and apolipoprotein C-I' to be significantly more abundant in the non-responsive patients compared to the responsive patients 24-hours post drug administration. The results suggest that apolipoprotein C-I and apolipoprotein C-I' may be used as early biomarkers for bortezomib drug resistance. However, it will be necessary to carry out a time course experiment in a larger sample size in order to validate these findings. Additional experiments are required to determine the functional relationship between these proteins and bortezomib response.

#### Dexamethasone

Dexamethasone (dex) is a synthetic steroid hormone that is also used in the treatment of MM. Clinical trials have shown response rates of up to 70% in MM patients [76]. Additional clinical trials observed a synergistic response when dex was used in combination with other drugs such as bortezomib and thalidomide [68,77]. The mechanisms of action of these drugs, however, are not well understood. In an attempt to improve clinical response Ress-Unwin *et al*. used mass spectrometry to identify proteins that may play a role in dex induced apoptosis [76]. They found a panel of proteins that were differentially expressed following dex treatment in the sensitive MM1S multiple myeloma cell line compared to the resistant MM1R cell line. Most notably, they identified FK binding protein 5 (FKBP5), which is involved in protein folding and trafficking to be over-expressed in the MM1S but not the MM1R cell line following dex treatment. These data are important as they shed light onto the signaling pathways that may induce dex-mediated apoptosis and thus may help direct rational drug design. However, before this is realized it will be necessary to gain further insight into the signaling pathways in which these proteins are acting.

#### Arsenic trioxide

Arsenic trioxide (ATO) has been shown to induce growth inhibition and apoptosis in MM cells and has shown clinical activity in both Phase I and II clinical trials in patients with relapsed or refractory MM [78]. In order to determine the mechanisms of ATO activity, Ge *et al*. used 2D gel electrophoresis coupled with MALDI TOF/TOF analysis to evaluate proteins altered following ATO activity in the U266 multiple myeloma cell line [78]. The most significant changes were observed in the up-regulation of HSP proteins and down regulation of 14-3-3ζ protein and the members of the ubiquitin-proteasome system following ATO treatment. This group further demonstrated that the use of 14-3-3ζ siRNA potentiated the effects of ATO induced apoptosis whereas over-expression 14-3-3ζ reduced ATO-sensitivity in U266 cells. Furthermore, they showed that inhibition of HSP90, which is over-expressed following ATO treatment, sensitized cells to ATO treatment as well as potentiated the effect of 14-3-3ζ knockdown. These results demonstrate the usefulness of identifying additional therapeutic targets that may be exploited to over-come drug resistance.

### Post translation modifications of the MM proteome

Post-translation modifications (PTMs) play an important role in the maturation and regulation of proteins. One of the most common PTMs is phosphorylation. Phosphorylation of proteins is carried out by specific protein kinases and occurs at three specific residues: serine, threonine, and tyrosine. Protein dephosphorylation, on the other hand is carried out by phosphatases. Protein activity is controlled by cycles of phosphorylation and dephosphorylation. Because protein phosphorylation is crucial to protein activity and thus regulation of cellular behavior, knowledge of protein phosphorylation status within the cell would give significant insight into signaling mechanisms. Furthermore, this may help in the design of kinase or phosphatase inhibitors in an attempt to control cellular events.

In MM, several proteins are regulated through phosphorylation events, including fibroblast growth factor receptor-3 (FGFR3). Activation of FGFR3, through tyrosine phosphorylation, induces cell growth, survival and migration through activation of various signaling pathways including MAPK and PI3K [79,80]. Aberrant activation of FGFR3 has been observed in 15-20% of MM due to a t(4;14)(p16.3;q32) translocation and has been shown to contribute to the tumorgenesis of MM, including chemoresistance [81-83]. For these reasons several drugs have been designed to target this receptor [84].

The signaling networks activated downstream of FGFR3 are not fully known. Recently, St-Germain *et al*. studied the phosphotyrosine proteomic profile associated with FGFR3 expression, ligand activation, and drug inhibition in the KMS11 MM cell line by mass spectrometry [85]. They identified and quantified several phosphotyrosine sites as a result of FGFR3 activation and drug inhibition. Their results have substantially increased our understanding of FGFR3 function and provided a framework for studying appropriate signaling networks activated by this receptor in MM. Importantly their mass spectrometry approach demonstrated the potential for pharmacodynamic monitoring.

## The future of mass spectrometry in biomarker discovery

The use of mass spectrometry for biomarker discovery holds great promise. In order for this to be fully realized in the clinical setting however, various limitations must be addressed [62]. First, there exists a limited dynamic range for even the most sensitive mass spectrometers. Highly abundant proteins, such as albumin can mask less abundant proteins which may be important biomarkers. This is especially true during the early stages of tumor development when tumor biomarkers may be low and so care must be taken to simplify samples. Through sample purification, however, low-abundant proteins maybe lost through interactions with high abundant proteins such as albumin. Thus, all purifications steps should be analyzed.

It will also be important that biomarkers are validated in large, independent studies before entering the clinic. To this end, it will be necessary to standardize these experiments in terms of sample collection, storage and processing as well as bio-informatics and statistical analysis between various centers. Furthermore, careful consideration will need to be given to the normal group to which the cancer group is compared. Differences in age, sex, and ethnicity, as well as menopausal and nutritional status may all be confounding variables in biomarker discovery [86,87].

Although both cell lines and clinical specimens are valuable samples for biomarker discovery, they each have their limitations. Most notably, cell lines do not represent an in vivo model. Therefore, the influence of the microenvironment on the tumor biomarker signature cannot be evaluated resulting in potential misrepresentation of the true biomarkers. In terms of clinical specimens, as discussed above, the many confounding variable associated when comparing tumor to normal tissue also represents a hurdle impeding biomarker discovery. An alternative to these approaches is the use of genetically engineered mouse models. These mouse models offer the opportunity to standardize experiments through homogenized breeding and environmental control and by defining stages of tumor development [64]. Importantly it has been observed that the tumor antigen repertoire of tumor-bearing transgenic mice can predict human tumor antigens [88].

## Conclusion

The use of mass spectral analysis will prove to be a valuable tool for the diagnosis, prognosis and response to drug treatment in cancer. Studies that have been carried out in MM have increased our understanding of this cancer; they have identified new serum biomarkers that may distinguish between MM and normal patients as well as serum markers that may identify patients with skeletal involvement. In addition, mass spectrometry has been used to identify biomarkers that indicate resistance to several chemotherapeutic drugs used to treat MM. Equally important mass spectrometry was used to investigate the phosphotyrosine signaling pathways downstream of FGFR3. These types of studies, that investigate signaling networks, are essential as they will help guide future investigations into the pathogenesis of MM.

Perhaps one of the greatest promises of mass spectrometry will be its use in helping direct therapy. Since current "one size fits all" therapy is complicated by serious toxicities and may be unnecessary in some good prognosis patients, it is critical to introduce risk-adapted therapy. The development of risk-adapted therapy requires better prognostic markers as the current prognostic models remain inadequate to predict disease outcome for individual patients. Through protein expression profiling by mass spectrometry we will be able to identify biomarkers that can be used to improve the diagnosis and prognosis of MM as well as increase our understanding of the mechanisms of drug resistance, which will help direct therapeutic strategies.

## Competing interests

The authors declare that they have no competing interests.

## Authors' contributions

JM and HC drafted manuscript; JM, MD, JC, SA, KV, LQ and HC participated in the design and analysis of the MM proteomic data described in the manuscript; all contributed to the critical revision of the manuscript. HC supervised the study, provided funding and approved the final manuscript. All authors read and approved the final manuscript.

## References

[B1] Raab MS Podar K Breitkreutz I Richardson PG Anderson KC Multiple myeloma Lancet 2009 374 324 339 10.1016/S0140-6736(09)60221-X 19541364

[B2] Bergsagel PL Chesi M Nardini E Brents LA Kirby SL Kuehl WM Promiscuous translocations into immunoglobulin heavy chain switch regions in multiple myeloma Proc Natl Acad Sci USA 1996 93 13931 13936 10.1073/pnas.93.24.13931 8943038 PMC19472

[B3] Bergsagel PL Kuehl WM Chromosome translocations in multiple myeloma Oncogene 2001 20 5611 5622 10.1038/sj.onc.1204641 11607813

[B4] Bergsagel PL Nardini E Brents L Chesi M Kuehl WM IgH translocations in multiple myeloma: a nearly universal event that rarely involves c-myc Curr Top Microbiol Immunol 1997 224 283 287 9308253 10.1007/978-3-642-60801-8_30

[B5] Barille-Nion S Barlogie B Bataille R Bergsagel PL Epstein J Fenton RG Jacobson J Kuehl WM Shaughnessy J Tricot G Advances in biology and therapy of multiple myeloma Hematology Am Soc Hematol Educ Program 2003 248 278 14633785

[B6] Onwuazor ON Wen XY Wang DY Zhuang L Masih-Khan E Claudio J Barlogie B Shaughnessy JD Jr Stewart AK Mutation, SNP, and isoform analysis of fibroblast growth factor receptor 3 (FGFR3) in 150 newly diagnosed multiple myeloma patients Blood 2003 102 772 773 10.1182/blood-2003-04-1204 12835230

[B7] Fonseca R Bailey RJ Ahmann GJ Rajkumar SV Hoyer JD Lust JA Kyle RA Gertz MA Greipp PR Dewald GW Genomic abnormalities in monoclonal gammopathy of undetermined significance Blood 2002 100 1417 1424 12149226

[B8] Kaufmann H Ackermann J Baldia C Nosslinger T Wieser R Seidl S Sagaster V Gisslinger H Jager U Pfeilstocker M Both IGH translocations and chromosome 13q deletions are early events in monoclonal gammopathy of undetermined significance and do not evolve during transition to multiple myeloma Leukemia 2004 18 1879 1882 10.1038/sj.leu.2403518 15385925

[B9] Kuehl WM Bergsagel PL Multiple myeloma: evolving genetic events and host interactions Nat Rev Cancer 2002 2 175 187 10.1038/nrc746 11990854

[B10] Chang H Bartlett ES Patterson B Chen CI Yi QL The absence of CD56 on malignant plasma cells in the cerebrospinal fluid is the hallmark of multiple myeloma involving central nervous system Br J Haematol 2005 129 539 541 10.1111/j.1365-2141.2005.05493.x 15877737

[B11] Chang H Bouman D Boerkoel CF Stewart AK Squire JA Frequent monoallelic loss of D13S319 in multiple myeloma patients shown by interphase fluorescence in situ hybridization Leukemia 1999 13 105 109 10.1038/sj/leu/2401208 10049044

[B12] Chang H Li D Zhuang L Nie E Bouman D Stewart AK Chun K Detection of chromosome 13q deletions and IgH translocations in patients with multiple myeloma by FISH: comparison with karyotype analysis Leuk Lymphoma 2004 45 965 969 10.1080/10428190310001638832 15291356

[B13] Chang H Ning Y Qi X Yeung J Xu W Chromosome 1p21 deletion is a novel prognostic marker in patients with multiple myeloma Br J Haematol 2007 139 51 54 10.1111/j.1365-2141.2007.06750.x 17854306

[B14] Chang H Qi C Yi QL Reece D Stewart AK p53 gene deletion detected by fluorescence in situ hybridization is an adverse prognostic factor for patients with multiple myeloma following autologous stem cell transplantation Blood 2005 105 358 360 10.1182/blood-2004-04-1363 15339849

[B15] Chang H Qi X Trieu Y Xu W Reader JC Ning Y Reece D Multiple myeloma patients with CKS1B gene amplification have a shorter progression-free survival post-autologous stem cell transplantation Br J Haematol 2006 135 486 491 10.1111/j.1365-2141.2006.06325.x 16995883

[B16] Chang H Qi XY Claudio J Zhuang L Patterson B Stewart AK Analysis of PTEN deletions and mutations in multiple myeloma Leuk Res 2006 30 262 265 10.1016/j.leukres.2005.07.008 16112193

[B17] Chang H Qi XY Samiee S Yi QL Chen C Trudel S Mikhael J Reece D Stewart AK Genetic risk identifies multiple myeloma patients who do not benefit from autologous stem cell transplantation Bone Marrow Transplant 2005 36 793 796 10.1038/sj.bmt.1705131 16113669

[B18] Chang H Sloan S Li D Keith Stewart A Multiple myeloma involving central nervous system: high frequency of chromosome 17p13.1 (p53) deletions Br J Haematol 2004 127 280 284 10.1111/j.1365-2141.2004.05199.x 15491286

[B19] Chang H Sloan S Li D Patterson B Genomic aberrations in plasma cell leukemia shown by interphase fluorescence in situ hybridization Cancer Genet Cytogenet 2005 156 150 153 10.1016/j.cancergencyto.2004.05.004 15642395

[B20] Chang H Sloan S Li D Zhuang L Yi QL Chen CI Reece D Chun K Keith Stewart A The t(4;14) is associated with poor prognosis in myeloma patients undergoing autologous stem cell transplant Br J Haematol 2004 125 64 68 10.1111/j.1365-2141.2004.04867.x 15015970

[B21] Chang H Stewart AK Qi XY Li ZH Yi QL Trudel S Immunohistochemistry accurately predicts FGFR3 aberrant expression and t(4;14) in multiple myeloma Blood 2005 106 353 355 10.1182/blood-2005-01-0033 15761022

[B22] Chang H Yeung J Qi C Xu W Aberrant nuclear p53 protein expression detected by immunohistochemistry is associated with hemizygous P53 deletion and poor survival for multiple myeloma Br J Haematol 2007 138 324 329 10.1111/j.1365-2141.2007.06649.x 17555471

[B23] Chang H Yeung J Xu W Ning Y Patterson B Significant increase of CKS1B amplification from monoclonal gammopathy of undetermined significance to multiple myeloma and plasma cell leukaemia as demonstrated by interphase fluorescence in situ hybridisation Br J Haematol 2006 134 613 615 10.1111/j.1365-2141.2006.06237.x 16889615

[B24] Chang H Qi XY Stewart AK t(11;14) does not predict long-term survival in myeloma Leukemia 2005 19 1078 1079 10.1038/sj.leu.2403744 15815719

[B25] Chang H Qi X Jiang A Xu W Young T Reece D 1p21 deletions are strongly associated with 1q21 gains and are an independent adverse prognostic factor for the outcome of high-dose chemotherapy in patients with multiple myeloma Bone Marrow Transplant 2009 45 1 117 21 10.1038/bmt.2009.107 19448682

[B26] Jaksic W Trudel S Chang H Trieu Y Qi X Mikhael J Reece D Chen C Stewart AK Clinical outcomes in t(4;14) multiple myeloma: a chemotherapy-sensitive disease characterized by rapid relapse and alkylating agent resistance J Clin Oncol 2005 23 7069 7073 10.1200/JCO.2005.17.129 16129840

[B27] Chang H Trieu Y Qi X Xu W Stewart KA Reece D Bortezomib therapy response is independent of cytogenetic abnormalities in relapsed/refractory multiple myeloma Leuk Res 2007 31 779 782 10.1016/j.leukres.2006.08.002 16996589

[B28] Reece D Song KW Fu T Roland B Chang H Horsman DE Mansoor A Chen C Masih-Khan E Trieu Y Influence of cytogenetics in patients with relapsed or refractory multiple myeloma treated with lenalidomide plus dexamethasone: adverse effect of deletion 17p13 Blood 2009 114 522 525 10.1182/blood-2008-12-193458 19332768

[B29] Mulligan G Mitsiades C Bryant B Zhan F Chng WJ Roels S Koenig E Fergus A Huang Y Richardson P Gene expression profiling and correlation with outcome in clinical trials of the proteasome inhibitor bortezomib Blood 2007 109 3177 3188 10.1182/blood-2006-09-044974 17185464

[B30] Chauhan D Auclair D Robinson EK Hideshima T Li G Podar K Gupta D Richardson P Schlossman RL Krett N Identification of genes regulated by dexamethasone in multiple myeloma cells using oligonucleotide arrays Oncogene 2002 21 1346 1358 10.1038/sj.onc.1205205 11857078

[B31] Watts GS Futscher BW Isett R Gleason-Guzman M Kunkel MW Salmon SE cDNA microarray analysis of multidrug resistance: doxorubicin selection produces multiple defects in apoptosis signaling pathways J Pharmacol Exp Ther 2001 299 434 441 11602652

[B32] Davies FE Dring AM Li C Rawstron AC Shammas MA O'Connor SM Fenton JA Hideshima T Chauhan D Tai IT Insights into the multistep transformation of MGUS to myeloma using microarray expression analysis Blood 2003 102 4504 4511 10.1182/blood-2003-01-0016 12947006

[B33] Ideker T Thorsson V Ranish JA Christmas R Buhler J Eng JK Bumgarner R Goodlett DR Aebersold R Hood L Integrated genomic and proteomic analyses of a systematically perturbed metabolic network Science 2001 292 929 934 10.1126/science.292.5518.929 11340206

[B34] Gygi SP Rochon Y Franza BR Aebersold R Correlation between protein and mRNA abundance in yeast Mol Cell Biol 1999 19 1720 1730 10022859 10.1128/mcb.19.3.1720 PMC83965

[B35] Anderson L Seilhamer JAG A comparison of selected mRNA and protein abundances in human liver Electrophoresis 1997 18 533 537 10.1002/elps.1150180333 9150937

[B36] Micallef J Gajadhar A Wiley J DeSouza LV Michael Siu KW Guha A Proteomics: present and future implications in neuro-oncology Neurosurgery 2008 62 539 555 discussion 539-555 10.1227/01.neu.0000317302.85837.61 18425004

[B37] Liebler D Introduction to Proteomics 2002 Totowa, NJ: Humana Press

[B38] Zhao L Lee BY Brown DA Molloy MP Marx GM Pavlakis N Boyer MJ Stockler MR Kaplan W Breit SN Identification of candidate biomarkers of therapeutic response to docetaxel by proteomic profiling Cancer Res 2009 69 7696 7703 10.1158/0008-5472.CAN-08-4901 19773444

[B39] Bijian K Mlynarek AM Balys RL Jie S Xu Y Hier MP Black MJ Di Falco MR LaBoissiere S Alaoui-Jamali MA Serum proteomic approach for the identification of serum biomarkers contributed by oral squamous cell carcinoma and host tissue microenvironment J Proteome Res 2009 8 2173 2185 10.1021/pr800979e 19284786

[B40] Bouchal P Roumeliotis T Hrstka R Nenutil R Vojtesek B Garbis SD Biomarker discovery in low-grade breast cancer using isobaric stable isotope tags and two-dimensional liquid chromatography-tandem mass spectrometry (iTRAQ-2DLC-MS/MS) based quantitative proteomic analysis J Proteome Res 2009 8 362 373 10.1021/pr800622b 19053527

[B41] DeSouza LV Romaschin AD Colgan TJ Siu KW Absolute quantification of potential cancer markers in clinical tissue homogenates using multiple reaction monitoring on a hybrid triple quadrupole/linear ion trap tandem mass spectrometer Anal Chem 2009 81 3462 3470 10.1021/ac802726a 19323455

[B42] Zhu H Dale PS Caldwell CW Fan X Rapid and label-free detection of breast cancer biomarker CA15-3 in clinical human serum samples with optofluidic ring resonator sensors Anal Chem 2009 81 9858 9865 10.1021/ac902437g 19911811

[B43] Rower C Vissers JP Koy C Kipping M Hecker M Reimer T Gerber B Thiesen HJ Glocker MO Towards a proteome signature for invasive ductal breast carcinoma derived from label-free nanoscale LC-MS protein expression profiling of tumorous and glandular tissue Anal Bioanal Chem 2009 395 2443 2456 10.1007/s00216-009-3187-9 19876624

[B44] Fatima N Chelius D Luke BT Yi M Zhang T Stauffer S Stephens R Lynch P Miller K Guszczynski T Label-free global serum proteomic profiling reveals novel celecoxib-modulated proteins in familial adenomatous polyposis patients Cancer Genomics Proteomics 2009 6 41 49 19451089

[B45] Pan J Chen HQ Sun YH Zhang JH Luo XY Comparative proteomic analysis of non-small-cell lung cancer and normal controls using serum label-free quantitative shotgun technology Lung 2008 186 255 261 10.1007/s00408-008-9093-7 18465169

[B46] Griffin NM Yu J Long F Oh P Shore S Li Y Koziol JA Schnitzer JE Label-free, normalized quantification of complex mass spectrometry data for proteomic analysis Nat Biotechnol 2010 28 83 89 10.1038/nbt.1592 20010810 PMC2805705

[B47] Liu H Sadygov RG Yates JR A model for random sampling and estimation of relative protein abundance in shotgun proteomics Anal Chem 2004 76 4193 4201 10.1021/ac0498563 15253663

[B48] Gao J Opiteck GJ Friedrichs MS Dongre AR Hefta SA Changes in the protein expression of yeast as a function of carbon source J Proteome Res 2003 2 643 649 10.1021/pr034038x 14692458

[B49] Zhang B VerBerkmoes NC Langston MA Uberbacher E Hettich RL Samatova NF Detecting differential and correlated protein expression in label-free shotgun proteomics J Proteome Res 2006 5 2909 2918 10.1021/pr0600273 17081042

[B50] Old WM Meyer-Arendt K Aveline-Wolf L Pierce KG Mendoza A Sevinsky JR Resing KA Ahn NG Comparison of label-free methods for quantifying human proteins by shotgun proteomics Mol Cell Proteomics 2005 4 1487 1502 10.1074/mcp.M500084-MCP200 15979981

[B51] Zybailov B Coleman MK Florens L Washburn MP Correlation of relative abundance ratios derived from peptide ion chromatograms and spectrum counting for quantitative proteomic analysis using stable isotope labeling Anal Chem 2005 77 6218 6224 10.1021/ac050846r 16194081

[B52] Mueller LN Brusniak MY Mani DR Aebersold R An assessment of software solutions for the analysis of mass spectrometry based quantitative proteomics data J Proteome Res 2008 7 51 61 10.1021/pr700758r 18173218

[B53] Bantscheff M Schirle M Sweetman G Rick J Kuster B Quantitative mass spectrometry in proteomics: a critical review Anal Bioanal Chem 2007 389 1017 1031 10.1007/s00216-007-1486-6 17668192

[B54] Simpson KL Whetton AD Dive C Quantitative mass spectrometry-based techniques for clinical use: biomarker identification and quantification J Chromatogr B Analyt Technol Biomed Life Sci 2009 877 1240 1249 10.1016/j.jchromb.2008.11.023 19058768 PMC7185464

[B55] Landgren O Kyle RA Pfeiffer RM Katzmann JA Caporaso NE Hayes RB Dispenzieri A Kumar S Clark RJ Baris D Monoclonal gammopathy of undetermined significance (MGUS) consistently precedes multiple myeloma: a prospective study Blood 2009 113 5412 5417 10.1182/blood-2008-12-194241 19179464 PMC2689042

[B56] Ritchie SA Ahiahonu PW Jayasinghe D Heath D Liu J Lu Y Jin W Kavianpour A Yamazaki Y Khan AM Reduced levels of hydroxylated, polyunsaturated ultra long-chain fatty acids in the serum of colorectal cancer patients: implications for early screening and detection BMC Med 2010 8 13 10.1186/1741-7015-8-13 20156336 PMC2833138

[B57] Schaaij-Visser TB Brakenhoff RH Leemans CR Heck AJ Slijper M Protein biomarker discovery for head and neck cancer J Proteomics 2010 in press 20139037 10.1016/j.jprot.2010.01.013

[B58] Gromov P Gromova I Bunkenborg J Cabezon T Moreira JM Timmermans-Wielenga V Roepstorff P Rank F Celis JE Up-regulated proteins in the fluid bathing the tumour cell microenvironment as potential serological markers for early detection of cancer of the breast Mol Oncol 2010 4 65 89 10.1016/j.molonc.2009.11.003 20005186 PMC5527961

[B59] Kawase H Fujii K Miyamoto M Kubota KC Hirano S Kondo S Inagaki F Differential LC-MS-based proteomics of surgical human cholangiocarcinoma tissues J Proteome Res 2009 8 4092 4103 10.1021/pr900468k 19569727

[B60] Li G Zhang W Zeng H Chen L Wang W Liu J Zhang Z Cai Z An integrative multi-platform analysis for discovering biomarkers of osteosarcoma BMC Cancer 2009 9 150 10.1186/1471-2407-9-150 19445706 PMC2691408

[B61] Yee J Sadar MD Sin DD Kuzyk M Xing L Kondra J McWilliams A Man SF Lam S Connective tissue-activating peptide III: a novel blood biomarker for early lung cancer detection J Clin Oncol 2009 27 2787 2792 10.1200/JCO.2008.19.4233 19414677 PMC2698017

[B62] Davis MA Hanash S High-throughput genomic technology in research and clinical management of breast cancer. Plasma-based proteomics in early detection and therapy Breast Cancer Res 2006 8 217 10.1186/bcr1619 17184556 PMC1797031

[B63] Faca V Krasnoselsky A Hanash S Innovative proteomic approaches for cancer biomarker discovery Biotechniques 2007 43 279 281-273, 285 10.2144/000112541 17907570

[B64] Hanash SM Pitteri SJ Faca VM Mining the plasma proteome for cancer biomarkers Nature 2008 452 571 579 10.1038/nature06916 18385731

[B65] Koomen JM Haura EB Bepler G Sutphen R Remily-Wood ER Benson K Hussein M Hazlehurst LA Yeatman TJ Hildreth LT Proteomic contributions to personalized cancer care Mol Cell Proteomics 2008 7 1780 1794 10.1074/mcp.R800002-MCP200 18664563 PMC2559938

[B66] Wang QT Li YZ Liang YF Hu CJ Zhai YH Zhao GF Zhang J Li N Ni AP Chen WM Xu Y Construction of a multiple myeloma diagnostic model by magnetic bead-based MALDI-TOF mass spectrometry of serum and pattern recognition software Anat Rec (Hoboken) 2009 292 604 610 19301277 10.1002/ar.20871

[B67] Bhattacharyya S Epstein J Suva LJ Biomarkers that discriminate multiple myeloma patients with or without skeletal involvement detected using SELDI-TOF mass spectrometry and statistical and machine learning tools Dis Markers 2006 22 245 255 17124346 10.1155/2006/728296 PMC3862287

[B68] Richardson PG Barlogie B Berenson J Singhal S Jagannath S Irwin D Rajkumar SV Srkalovic G Alsina M Alexanian R A phase 2 study of bortezomib in relapsed, refractory myeloma N Engl J Med 2003 348 2609 2617 10.1056/NEJMoa030288 12826635

[B69] Jagannath S Barlogie B Berenson J Siegel D Irwin D Richardson PG Niesvizky R Alexanian R Limentani SA Alsina M A phase 2 study of two doses of bortezomib in relapsed or refractory myeloma Br J Haematol 2004 127 165 172 10.1111/j.1365-2141.2004.05188.x 15461622

[B70] Adams J The proteasome: a suitable antineoplastic target Nat Rev Cancer 2004 4 349 360 10.1038/nrc1361 15122206

[B71] Jagannath S Richardson PG Sonneveld P Schuster MW Irwin D Stadtmauer EA Facon T Harousseau JL Cowan JM Anderson KC Bortezomib appears to overcome the poor prognosis conferred by chromosome 13 deletion in phase 2 and 3 trials Leukemia 2007 21 151 157 10.1038/sj.leu.2404442 17096017

[B72] Sagaster V Ludwig H Kaufmann H Odelga V Zojer N Ackermann J Kuenburg E Wieser R Zielinski C Drach J Bortezomib in relapsed multiple myeloma: response rates and duration of response are independent of a chromosome 13q-deletion Leukemia 2007 21 164 168 10.1038/sj.leu.2404459 17096015

[B73] Micallef JCJ Dharsee M Ackloo S Evans K Chang H-bomb Elucidation of Proteins Involved in the Bortezomib Resistance of Multiple Myelomas though iTRAQ Analysis Book Elucidation of Proteins Involved in the Bortezomib Resistance of Multiple Myelomas though iTRAQ Analysis (Editor). City 2010

[B74] Micallef J Taccone M Mukherjee J Croul S Busby J Moran MF Guha A Epidermal growth factor receptor variant III-induced glioma invasion is mediated through myristoylated alanine-rich protein kinase C substrate overexpression Cancer Res 2009 69 7548 7556 10.1158/0008-5472.CAN-08-4783 19773446

[B75] Hsieh FY Tengstrand E Pekol TM Guerciolini R Miwa G Elucidation of potential bortezomib response markers in mutliple myeloma patients J Pharm Biomed Anal 2009 49 115 122 10.1016/j.jpba.2008.09.053 19062221

[B76] Rees-Unwin KS Craven RA Davenport E Hanrahan S Totty NF Dring AM Banks RE G JM Davies FE Proteomic evaluation of pathways associated with dexamethasone-mediated apoptosis and resistance in multiple myeloma Br J Haematol 2007 139 559 567 10.1111/j.1365-2141.2007.06837.x 17979943

[B77] Richardson PG Schlossman RL Weller E Hideshima T Mitsiades C Davies F LeBlanc R Catley LP Doss D Kelly K Immunomodulatory drug CC-5013 overcomes drug resistance and is well tolerated in patients with relapsed multiple myeloma Blood 2002 100 3063 3067 10.1182/blood-2002-03-0996 12384400

[B78] Ge F Lu XP Zeng HL He QY Xiong S Jin L He QY Proteomic and functional analyses reveal a dual molecular mechanism underlying arsenic-induced apoptosis in human multiple myeloma cells J Proteome Res 2009 8 3006 3019 10.1021/pr9001004 19364129

[B79] Eswarakumar VP Lax I Schlessinger J Cellular signaling by fibroblast growth factor receptors Cytokine Growth Factor Rev 2005 16 139 149 10.1016/j.cytogfr.2005.01.001 15863030

[B80] L'Hote CG Knowles MA Cell responses to FGFR3 signalling: growth, differentiation and apoptosis Exp Cell Res 2005 304 417 431 10.1016/j.yexcr.2004.11.012 15748888

[B81] Trudel S Stewart AK Rom E Wei E Li ZH Kotzer S Chumakov I Singer Y Chang H Liang SB Yayon A The inhibitory anti-FGFR3 antibody, PRO-001, is cytotoxic to t(4;14) multiple myeloma cells Blood 2006 107 4039 4046 10.1182/blood-2005-10-4179 16467200

[B82] Chesi M Nardini E Lim RS Smith KD Kuehl WM Bergsagel PL The t(4;14) translocation in myeloma dysregulates both FGFR3 and a novel gene, MMSET, resulting in IgH/MMSET hybrid transcripts Blood 1998 92 3025 3034 9787135

[B83] Pollett JB Trudel S Stern D Li ZH Stewart AK Overexpression of the myeloma-associated oncogene fibroblast growth factor receptor 3 confers dexamethasone resistance Blood 2002 100 3819 3821 10.1182/blood-2002-02-0608 12393593

[B84] Paterson JL Li Z Wen XY Masih-Khan E Chang H Pollett JB Trudel S Stewart AK Preclinical studies of fibroblast growth factor receptor 3 as a therapeutic target in multiple myeloma Br J Haematol 2004 124 595 603 10.1111/j.1365-2141.2004.04814.x 14871245

[B85] St-Germain JR Taylor P Tong J Jin LL Nikolic A Stewart II Ewing RM Dharsee M Li Z Trudel S Moran MF Multiple myeloma phosphotyrosine proteomic profile associated with FGFR3 expression, ligand activation, and drug inhibition Proc Natl Acad Sci USA 2009 106 20127 20132 19901323 10.1073/pnas.0910957106 PMC2775037

[B86] Merwe DE van der Oikonomopoulou K Marshall J Diamandis EP Mass spectrometry: uncovering the cancer proteome for diagnostics Adv Cancer Res 2007 96 23 50 17161675 10.1016/S0065-230X(06)96002-3

[B87] Diamandis EP Analysis of serum proteomic patterns for early cancer diagnosis: drawing attention to potential problems J Natl Cancer Inst 2004 96 353 356 14996856 10.1093/jnci/djh056

[B88] Lu H Knutson KL Gad E Disis ML The tumor antigen repertoire identified in tumor-bearing neu transgenic mice predicts human tumor antigens Cancer Res 2006 66 9754 9761 10.1158/0008-5472.CAN-06-1083 17018635

[B89] Buzzeo R Enkemann S Nimmanapalli R Alsina M Lichtenheld MG Dalton WS Beaupre DM Characterization of a R115777-resistant human multiple myeloma cell line with cross-resistance to PS-341 Clin Cancer Res 2005 11 6057 6064 10.1158/1078-0432.CCR-04-2685 16115951

